# Physical Activity for People Using Mental Health Services: A Framework for Action in Wales

**DOI:** 10.1192/bjo.2026.11073

**Published:** 2026-06-30

**Authors:** Ollie John, Alka Ahuja, Joanna Dainton, Dafydd Huw

**Affiliations:** 1 Royal College of Psychiatrists Wales, Cardiff, United Kingdom; 2 NHS Wales, Cardiff, United Kingdom

## Abstract

**Aims:**

To evaluate the implementation and mental health outcomes of physical activity-based interventions within contexts specifically relevant to Wales.

We aimed to identify existing gaps in the literature, explore implications for local clinical practice, and provide a strategic solution for common mental health settings.

We sought to establish a framework for action and quality statements to embed physical activity within routine mental health care.

Prioritise action that will support the improvement of the physical health of people with severe and enduring mental health conditions, reducing the mortality gap between people who have severe and enduring mental health conditions and those that do not. This is inkeeping with aspirations of Welsh Government’s Mental Health and Wellbeing Strategy Delivery Plan 2025–2028.

**Methods:**

We utilised a multi-faceted methodology, including a structured mapping and narrative analysis of the broader international evidence base alongside a systematic review of Wales-specific evidence.

The systematic search followed PRISMA guidelines, targeting databases such as MEDLINE, PubMed, and PsycINFO using key terms like “mental illness”, “exercise”, and “Wales”.

Grey literature sources were also analysed to capture real-world practical challenges. Articles were screened, with inclusion limited to those published after 2007 to remain consistent with modern legislative definitions.

**Results:**

The findings indicate that physical activity interventions significantly improve mental wellbeing across various settings in Wales, including community-based programmes, secure units, and exercise referral schemes.

Key benefits identified include reduced symptom severity for both common mental disorders and severe mental illness (SMI), improved sleep quality, and mitigation of premature mortality risks.

However, significant barriers to implementation were identified:

• Systemic Barriers: Limited funding, a shortage of qualified physical activity professionals, and insufficient training for healthcare staff.

• Individual Barriers: Low motivation, poor baseline physical health, and low self-esteem among service users.

• Environmental Barriers: The “obesogenic” nature of secure inpatient units, which often lack the flexibility or resources to prioritize physical activity.

**Conclusion:**

Integrating physical activity into mental health services is essential but requires a shift in clinical culture and investment.

We propose four Quality Statements to guide leaders, emphasising collaborative design with physical activity professionals, inclusive programming for diverse needs, equitable care in secure settings, and sustainable investment in community programmes.

Effective implementation depends on moving toward a person-centred, evidence-informed approach that addresses both social and commercial determinants of health.

